# *De Novo* Assembly of Bitter Gourd Transcriptomes: Gene Expression and Sequence Variations in Gynoecious and Monoecious Lines

**DOI:** 10.1371/journal.pone.0128331

**Published:** 2015-06-05

**Authors:** Anjali Shukla, V. K. Singh, D. R. Bharadwaj, Rajesh Kumar, Ashutosh Rai, A. K. Rai, Raja Mugasimangalam, Sriram Parameswaran, Major Singh, P. S. Naik

**Affiliations:** 1 Division of Crop Improvement, Indian Institute of Vegetable Research, Post Bag No- 01, Jakhini (Shahanshahpur), Varanasi, Uttar Pradesh-221 305, India; 2 Centre for Bioinformatics, School of Biotechnology, Banaras Hindu University, Varanasi, Uttar Pradesh 221 005, India; 3 Centre of Advanced Studies, Department of Botany, Banaras Hindu University, Varanasi, Uttar Pradesh 221 005, India; 4 Genotypic Technology (P) Ltd., Bangalore 560094, Karnataka, India; National Institute of Plant Genome Research, INDIA

## Abstract

Bitter gourd (*Momordica charantia* L.) is a nutritious vegetable crop of Asian origin, used as a medicinal herb in Indian and Chinese traditional medicine. Molecular breeding in bitter gourd is in its infancy, due to limited molecular resources, particularly on functional markers for traits such as gynoecy. We performed *de novo* transcriptome sequencing of bitter gourd using Illumina next-generation sequencer, from root, flower buds, stem and leaf samples of gynoecious line (Gy323) and a monoecious line (DRAR1). A total of 65,540 transcripts for Gy323 and 61,490 for DRAR1 were obtained. Comparisons revealed SNP and SSR variations between these lines and, identification of gene classes. Based on available transcripts we identified 80 WRKY transcription factors, several reported in responses to biotic and abiotic stresses; 56 ARF genes which play a pivotal role in auxin-regulated gene expression and development. The data presented will be useful in both functions studies and breeding programs in bitter gourd.

## Introduction

Bitter gourd (*Momordica charantia* L., 2n = 2x = 22) is a cucurbitaceous vegetable originated in tropical Asia and is intensively distributed in India, China, Japan, Southeast Asia and many regions of Africa and South America. The exact information about its centre of origin, yet undefined, however, molecular studies indicate the centre of origin as areas within eastern India [1, 2, 3]. Bitter gourd also known as bitter melon, balsam apple, balsam pear, bitter squash, etc. and has been cultivated as food and medicines. The prefix ‘bitter’ to this crop has been most likely attributed to the compounds imparting the bitter taste. The important component of bitter gourd that manifests the medicinal properties are triterpine, phenolic compounds [4], momordicine [5], polypeptide-p [6], and has been rightly named as ‘cornucopia of health’ [7], with recent studies implicated mode of action for cancer cell suppression activity [8,9]. Apart from culinary preparations, bitter gourd is used in making sliced chips, herbal decoctions and in many other forms as ethno-medicines [10–12].

Bitter gourd is tropical flowering vine crop bearing solitary male and female flowers in the leaf axils. Monoecious (staminate and pistillate flowers on same plant) form of sex expression is predominant in bitter gourd [13], however, existence of gynoecious sex form (only pistillate flowers on a plant) has also been reported [14–17]. Regulation of sexual charterers in related cucurbits; melon (*C*. *melo*) and cucumber (*C*. *sativus*), has been known to be modulated by ethylene [18,19]. More recently ethylene biosynthesis had been directly linked to andromonoecy in melon [20]. Use of molecular breeding techniques in bitter gourd is in its infancy except for few molecular analyses for defining genetic diversity. However the genetic relation and conservation of response is less characterized. Recently a RAD-seq (restriction-associated DNA tag sequencing) analysis was used to reveal genome wide DNA polymorphisms and to genotype the F_2_ progeny from a cross between OHB61-5 (gynoecious line) and a monoecious line to identify DNA markers for gynoecy trait [21]. Conserved markers between cucurbits have been implicated in studies with larger scale characterization of molecular markers in related sponge gourd, that may be applied to bitter gourd [22]. A limited amount of transcript information (~14,000) have been documented for bitter gourd, using 454 sequencing, restricted to mining of unusual fatty acid biosynthesis pathways [23]. We present a comprehensive *de novo* transcriptome assembly of the bitter gourd for monoecious and gyneocious lines, and report a set of differentially expressed transcripts implicated in the floral differentiation, and demonstrate a set of transcripts annotated to the plant hormone response pathway that are significantly differentially regulated between the Gyno versus the Mono lines.

## Methods

### Sample Collection, RNA-Seq Library Preparation and Sequencing

Two accessions of bitter gourd, gynoecious (Gy323) and monoecious (DRAR1) lines (hereafter referred as Gyno and Mono, respectively) developed at Indian Institute of Vegetable Research, Varanasi, were selected for transcriptome sequencing. The major sex form in bitter gourd is monoecious; however, gynoecious sex type has also been reported [13–17]. The exploitation of gynoecy is cost-effective and easier for harnessing hybrid vigour in several cucurbitaceous crops including bitter gourd that have high male: female sex ratio requiring manual pollination. Five seeds of each inbreds of Gyno and Mono samples were grown in a glasshouse to the blooming phase. Plant samples (shoot, root, flower buds and young leaves) each of Gyno and Mono lines were collected, washed in ice cold 95% ethanol chopped in 1–2 mm dice and re-suspended in 15 ml RNA*later* solution (Ambion Cat#7020). Samples were stored in 50 ml falcon screw cap vials at 4°C for 2–3 h to allow permeation of RNA*later* into cells and subsequently shifted to -80°C till shipment. Total RNA was extracted from the root, flower buds, stem and young leaf. The quantitative and qualitative estimation was performed using Nanodrop Spectrophotometer and Agilent Bioanalyzer, respectively. RNA samples with 260/280 ratios (range 1.9 to 2.1), 260/230 (range 2.0 to 2.5) and RIN (RNA integrity number) more than 8.0 were considered for library preparation.

### Sequencing and Quality Controls

Transcriptome library for sequencing was constructed as per the IlluminaTruSeq RNA library protocol, quantified with Nanodrop prior to quality analysis using High Sensitivity Bioanalyzer Chip (Agilent). Two cDNA libraries were generated using mRNASeq assay for transcriptome sequencing on Illumina Genome Analyzer II platform. One paired-end (PE) cDNA library was brought forth from the pooled total RNA of shoot, root, young leaf and flower buds in equal quantity and sequencing was performed in one lane to generate 72 bp PE reads. Raw reads quality was assessed using SeqQC V2.0 (Genotypic Technology, Bangalore). High quality (HQ) reads filtering, vector contaminated reads filtering, adapter trimming and low quality end trimming was done using SeqQC V2.0. Post-quality processing, a total of 61,390,804 number of raw reads, 31,826,714 (31.83 millions) number of HQ reads for monoecious and 29,564,090 (29.56 millions) number of HQ reads for gynoecious line were obtained. Total raw reads in FASTQ file size 14.62 GB for Gyno and 15.06 GB for Mono were obtained. Total number of reads were 32,946,510 (32.95 millions) for Gyno and 33,912,199 (33.91 millions) for Mono whereas total number of HQ bases were 2202.59542 Mb for Gyno and 2355.78336 Mb for Mono. Percentage of HQ bases was ~96% for both genotypes.

### *De novo* Transcriptome Assembly

*De novo* assembly of short reads using de Bruijin graph was performed with Velvet_1.1.07 and Oases_0.2.01. Velvet (version 1.1.07) was used for assembly of short reads using de Bruijn graph algorithm and Oases (version 0.2.01) was used for *de novo* assembly of short reads to obtain best transcript assembly results with raw data [24, 25]. Total filtered transcript contigs having >200bp (~54,667 for Gyno and ~51,324 for Mono) were deposited in TSA (Transcriptome Shotgun Assembly) submission portal of NCBI database. The primary accession numbers for Gyno and Mono were GANF00000000 and GANG00000000, respectively.

### Mapping of Sequence Reads onto Bitter Gourd Transcripts

All the reads from three experiments were mapped to the non-redundant set of transcripts to quantify the abundance of transcripts, the number of reads and reads per million (rpm) corresponding to each transcript were determined. In addition, the coverage of each transcript was normalized to the number of reads per kilo base per million (rpkm).

### GC Content Analysis, SSRs Identification and SNP Detection

GC content analysis was performed using the SSR Locator and MISA (MIcroSAtellite; http://pgrc.ipk-gatersleben.de/misa) was used for identification of SSRs. The repeats of mono-, di-, tri-, tetra-, penta- and hexa-nucleotide, as well as compound microsatellites were considered for analysis using SSR Locator [26]. The minimum repeat number was ten for mono nucleotide repeats, six for di-nucleotide and five for tri-, tetra-, penta- & hexa-nucleotide repeats. Maximal distance interrupting two SSR in a compound microsatellite was 100bp. For SNP detection bowtie2-2.0.0-beta5 and Samtools 0.1.7a tools were used for alignment and for variation study [27–29]. Read depth > = 5X criteria was applied to call SNPs and in-dels.

### Similarity Search and Functional Annotation

Database annotation and match to available plant sequences of (non-redundant UniGene datasets from various species, including *Glycine max*, *Medicago truncatula*, *Lotus japonicus*, *Vigna unguiculata* and *Pisum sativum*), were performed using BLASTX and TBLASTX [30]. To derive the predicted functional annotation of *Momordica* transcripts, UniProt non-redundant protein and TAIR (The *Arabidopsis* Information Resource) data sets were used [31, 32]. BLAST hit with an E-value_1E205 was considered. The GO Slim terms for molecular function, biological process, and cellular component categories associated with the best BLASTX hit with *Arabidopsis* proteins were assigned to the corresponding *Momordica* transcript [33]. UniGene computationally identifies transcripts from the same locus and analyzes expression by tissue, age, and health condition. Eukaryotic Orthologous Groups (KOG) is a eukaryote-specific variant of the Clusters of Orthologous Groups (COG) tool that was applied for detection of ortholog and paralog proteins [34, 35].

### Transcription Factor Associated Gene Identification and Pathway Analysis

TBLASTN (Search translated nucleotide database using a protein query) was used for sequence alignment against Transcriptome Shotgun Assembly (TSA) of *Momordica charantia* (taxid: 3673) organism. The Arabidopsis transcription factor database sequences were used as model sequence for search against Transcriptome Shotgun Assembly (TSA) of *M*. *charantia*. *Dof* Transcription Factor associated genes were selected for phylogenetic analysis within and different plant species [36, 37]. All identified transcription factors were reported in MCTF Database for *M*. *charantia* Transcription Factors (http://www.insilicogenomics.in/mctdf/mctfd.html). For multiple sequence alignment and phylogenetic construction, ClustalW server was used [38, 39]. For pathway detection KAAS (KEGG Automatic Annotation Server: http://www.genome.jp/kegg/kaas/) server was used [40].

### RNA extraction and cDNA synthesis

Total RNA was extracted from bitter gourd plants from auxiliary branches having flower buds using the Trizol and following the manufacturer’s instructions (Invitrogen). To remove genomic DNA, the total RNA was digested with RNase-free DNaseI (Promega, USA) according to manufacturer’s recommendations. 1.2 μg of total RNA was used for preparation of first strand cDNA, using iScript cDNA Synthesis Kit (Bio-Rad Laboratories) following the manufacturer's protocol.

### Quantitative Real-Time PCR

Quantitative real-time PCR (qRT-PCR) was performed using the SsoFast EvaGreen Supermix RT-PCR kit (Bio-Rad Laboratories) and the iQ5 Thermal Cycler (Bio-Rad Laboratories, USA). The PCR mix was composed of 10 μl EvaGreen Supermix, 2.0 μl of 1:4 diluted cDNA, 0.5 μl of each primer (10 mM), and 7.5μl water in a final volume of 20 μl. The reactions were incubated under following cycling conditions: 2 min at 50°C, 2 min at 95°C, 40 cycles of 95°C for 30s, 56°C for 30s, and 72°C for 30s, and finally 72°C for 2 min with a single melt cycle from 65 to 95°C. Each sample was analysed in triplicate, and the expression levels were calculated using the 2-^ΔΔ^Ct comparative CT method [41]. Three independent experiments were performed. The primers used in qPCR are listed in S1 Table.

## Results

### Sequencing of Bitter gourd Transcriptome and *de novo* Assembly

A total of 4,509,781,854 raw reads in gynoecious pool and 4,759,081,108 in monoecious pool derived from root, flower buds, stem and leaf tissues were used for the *de novo* transcriptome assembly. The gynoecious line (Gy323) which bears only pistillate flowers, while the monoecious plant (DRAR1), bears both pistillate and staminate flowers. Inheritance of gynoecism (femaleness) has been documented in bitter gourd [42] and gynoecious lines are commercially used for cost effective hybrid seed production [43, 44]. Post quality filtering for low quality regions, adaptors and sequencing tags, a total read count of 65,056,390 reads for gynoecious and 67,509,182 reads for monoecious line were withdrawn for further processing. The matched reads found in Gy323 were 61,541,555 and 64,251,379 from DRAR1. On the basis of percentage of HQ bases best results from 15.0 GB monoecious (DRAR1) and 14.6 GB gynoecious (Gy323) FASTQ files were picked out for the *de novo* assembly. See primary report in materials and methods and S1 File.

*De novo* transcriptome assembly unlike genome assemblies, has been computationally challenging with short reads [45]. Current method rely on application of graph based assemblers that apply multiple k-mer optimization to handle alternate splice variant as well as deal with variable coverage [45, 46]. Velvet and Oasis are de Bujirin graph bases assemblers that have been applied to assemble transcripts from short read sequences [47]. To assemble the bitter gourd transcripts untrimmed high-quality sequence reads were assembled using Velvet program at k-mer length of 41(optimized using k-mer Genie) [48]. Oases program [46], *de novo* assembly of transcriptomes with short reads generated by Velvet as input was obtained to produce transcript isoforms. We performed assembly of contigs generated by Velvet for trimming dataset (k = 41) into transcripts using Oases with default parameters. This resulted in a total number of 127,026 transcripts (>100bp in length) (Table 1). The best assembly results were obtained with the second trimmed dataset. A total number of 69,980 contigs (>500bp in length) with a median length ~1,557 bp were generated (Table 1) that were considered further for annotation.

**Table 1:** Transcriptome *De novo* assembly statistics obtained from Velvet and Oases assembly.

Sample Name	Mono Pool		Gyno Pool	
Tool used	Velvet	Oases	Velvet	Oases
Hash length	41	NA	41	NA
**Contigs Generated**	30,092	61,490	34,086	65,540
**Maximum Contig Length**	9,160	10,413	11,022	11,022
**Minimum Contig Length**	100	100	100	100
**Average Contig Length**	885.424	919.403	812.298	904.721
**Total Contigs Length**	26,644,183	56,534,088	27,687,983	59,295,384
**Total Number of Non-ATGC Characters**	4,891	327	6136	261
**Percentage of Non-ATGC Characters**	0.000183567	5.78412e-06	0.00022161	4.40169e-06
**Contigs> 100 b**	30,055	61,488	34,040	65,538
**Contigs> 500 b**	16,559	34,187	17,244	35,793
**Contigs> 1 Kb**	9,686	20,738	9,540	21,450
**Contigs> 10 Kb**	0	1	1	1
**Contigs> 1 Mb**	0	0	0	0
**N50 value**	1,479	1,557	1,378	1,535
**No. reads assembled**	52,238,263	61,048,658	50,182,884	56,277,923
**Total no. of reads**	63,653,428	63,653,428	59,128,180	59,128,180
**% of reads assembled**	82.07%	95.91%	84.87%	95.18%

### Bitter Gourd Transcriptome Annotation

To identify the functional diversity and obtain insights into the complexity of the bitter gourd transcritpome, comprehensive annotation of the assembled transcripts was performed against non-redundant data sets. UniProt and UniGene data sets derived from plant species such as *Cucumis sativus*, *Vitis vinifera*, *Ricinius communis*, *Glycine max*, *Cucumis melo*, etc, were utilized in the analysis. The transcripts of bitter gourd were used for similarity search and sequence conservation against UniGene data sets of several species. The transcripts were matched to Uniprot KB Viridiplantae protein sequence datasets using BLAST. Contigs greater than 50% identity and 40% query coverage were considered to be suitable to assign annotation based on high degree of sequence identity. In accordance with this criteria maximal sequence level match was transcripts derived from *Cucumis sativus* followed by *Vitis*, *Ricinius*, *Populous*, *Medicago* and *Glycine*.

To get insight into the functional classes of genes identified between the Mono line (DRAR1) and the Gyno line (Gy323), a sub-set of 61,490 transcripts (of the 69,980 transcripts) were examined. Amongst these, 32,162 transcripts matched annotated database transcripts from NCBI, however 29,328 transcripts did not share significant sequence identity implicating novel signatures. Out of 32,162 annotated transcripts from Mono line, 6,987 numbers of proteins showed annotation to multiple transcripts and 11,518 transcripts showed significant similarity with sequences reported from *V*. *vinifera* followed by *R*. *communis* (6,925), *P*. *tricocarpa* (6,103), *M*. *truncatula* (1,408), and *A*. *thaliana* (347). For Gyno line Gy323, total 65,540 transcripts were examined. Among these, 33,758 transcripts showed similarities with annotated data while the unannotated transcripts were 31,782. Out of the total 33,758 annotated transcripts, 7,339 numbers of proteins showed annotation for more than one transcript. Maximum significant similarity for Gyno transcripts was with *V*. *vinifera* (11,893) followed by *R*. *communis* (7,113), *P*. *tricocarpa* (6,294), *M*. *truncatula* (1,560), and model plant *A*. *thaliana* (398). Based on sequence similarity search 99% identity was observed at minimum 40% query coverage with related organisms *C*. *lanatus*, *G*. *max*, *F*. *ananassa*, *C*. *melo*, *R*. *communis*, *B*. *vulgaris*, *B*. *pendula*, *G*. *wittmackii*, *V*. *vinifera*, *S*. *tuberosum* and *G*. *hirsutum*. For details of transcripts, see S2 and S3 Files.

### Functional Annotation and Characterization of Bitter Gourd Transcripts

**Fig 1:**
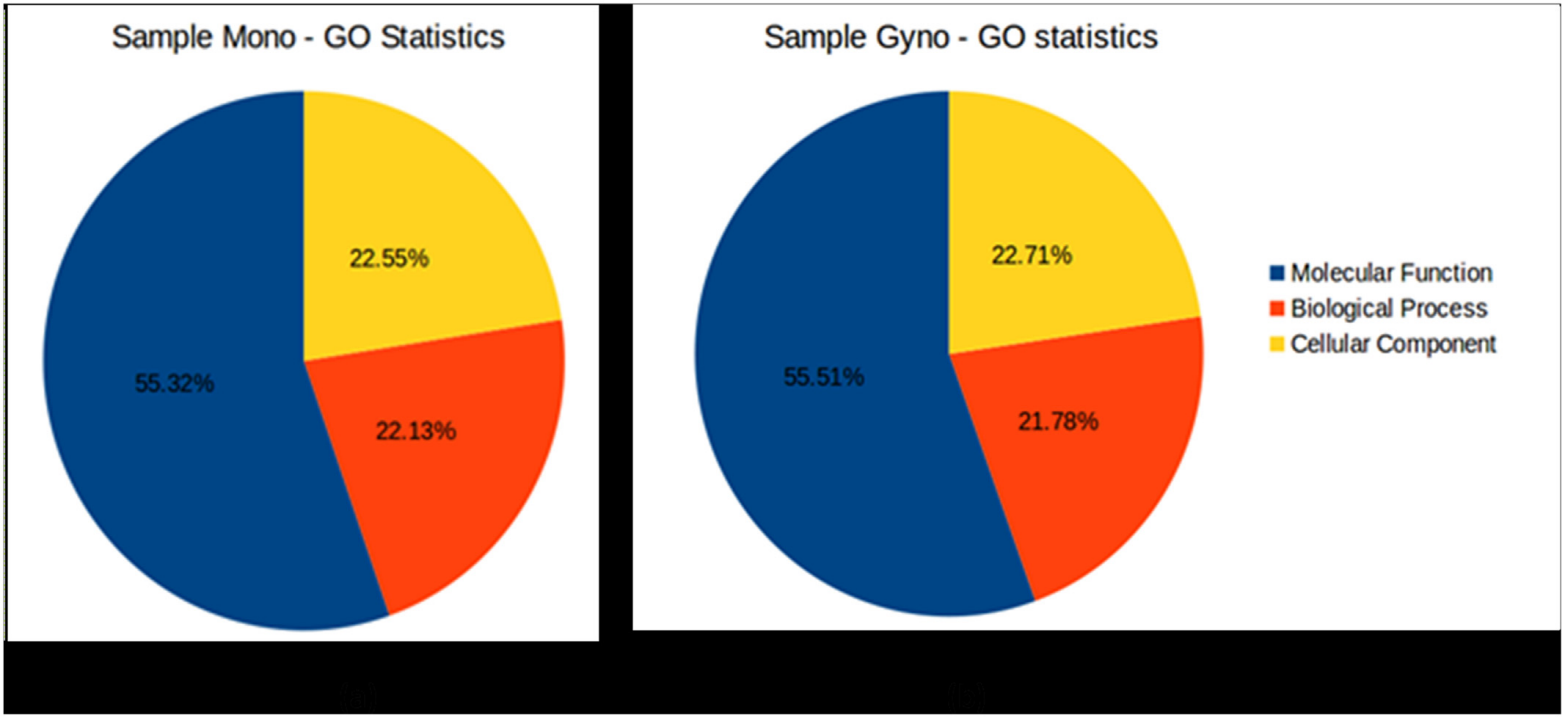
Molecular function, biological process and cellular component details statistics.

**Fig 2:**
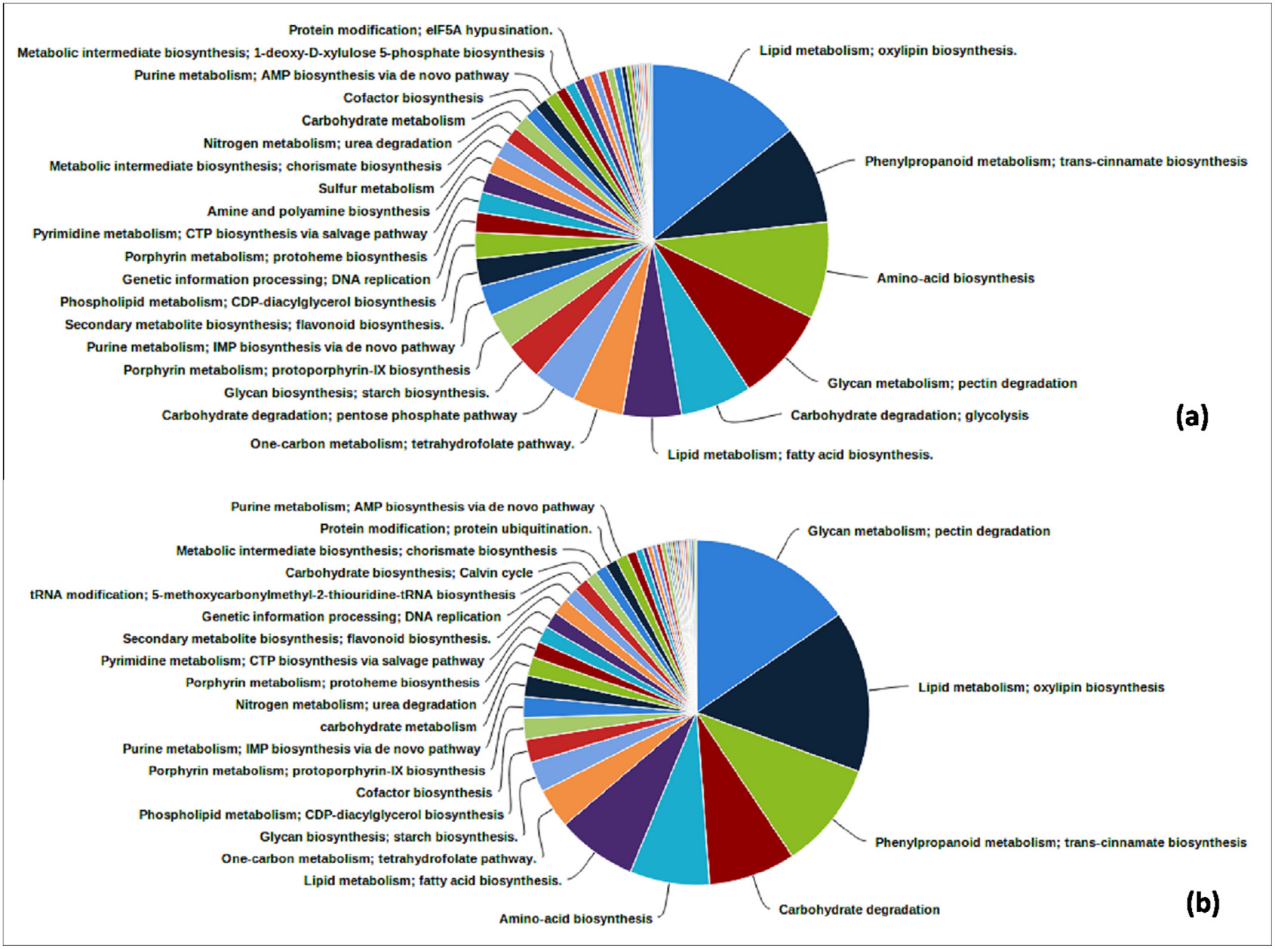
Detail statistics of identified pathways in bitter gourd transcripts.

We annotated 51.89% (65,920) transcripts of Mono and Gyno samples of the total 127,030 contigs. To detect the molecular functions, biological processes and cellular components, Gene Ontology (GO) database (AmiGO 2) was utilized to assign GO term for bitter gourd transcripts. Approximately 60% bitter gourd transcripts having GO terms, a total of 1,9229 (59.78%) transcripts of Mono was assigned at least one GO term among which all exhibit at least one GO term in molecular function, biological process and cellular component categories. For Gyno, 20,161 (59.72%) transcripts were assigned at least one GO term in molecular function, biological process and cellular component categories (Fig 1). Generally, the putative orthologs of genes involved in various pathways and cellular processes were found to be similar in bitter gourd (Fig 2, S3 File). Among the various biological processes, protein metabolism and developmental processes were highly represented compared to other biological process categories. For details, see S3 File.

### Unigene Identification and Pathway Analysis

**Fig 3:**
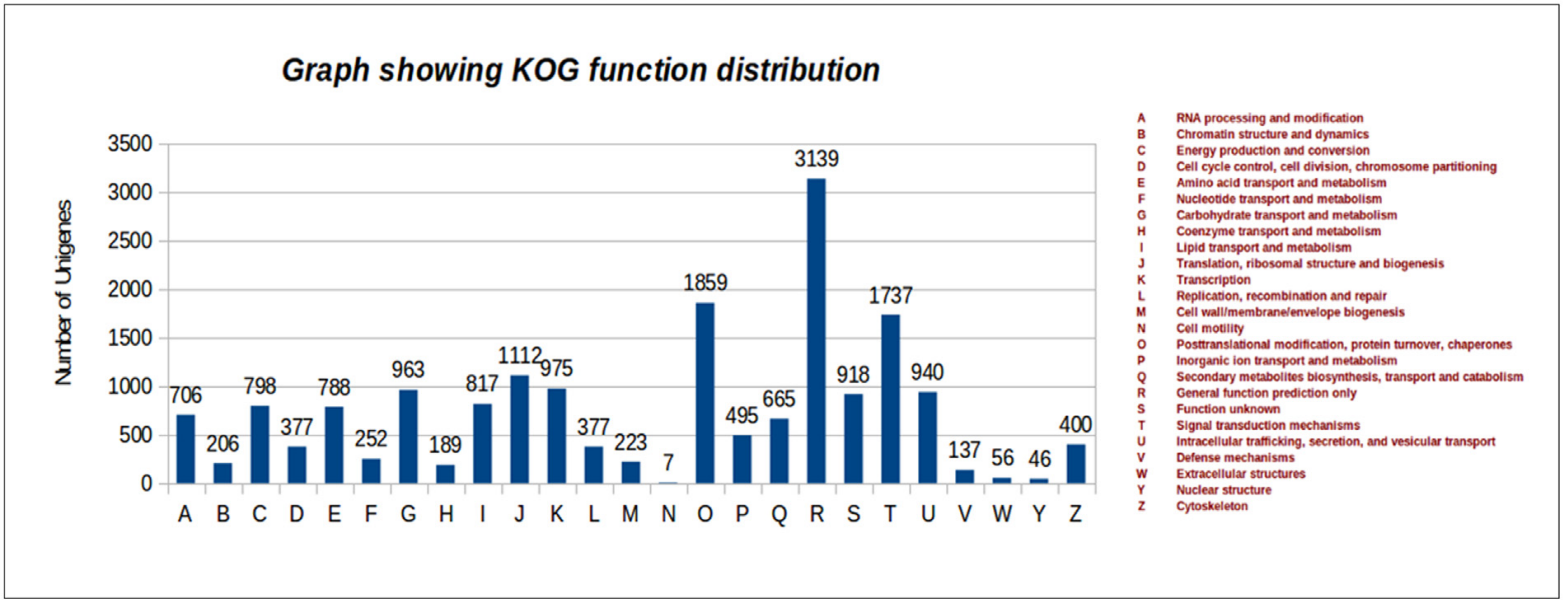
Graphical representation of KOG function distribution.

Based on KOG analysis the total number of ~36,000 unigenes for each genotype was found in transcriptome where around 13,000 unigenes annotated by KOG. About 37% of unigenes annotated by KOG database with cutoff 30% identity and 30% subject coverage. Function wise categorization was done and reported in S4 File. It was found that 137 unigenes annotated by KOG showed their role during defense mechanisms and nearly 400 transcripts involved in cytoskeleton development (Fig 3).

Identification of biochemical and cellular pathways were performed through the KAAS server, and resulted in 13,614 transcripts from the gynoecious line while 13,839 transcripts from the monoecious line having a role with specific pathways, for details, see S5 File.

### Identification of Transcription Factor Classes

**Fig 4:**
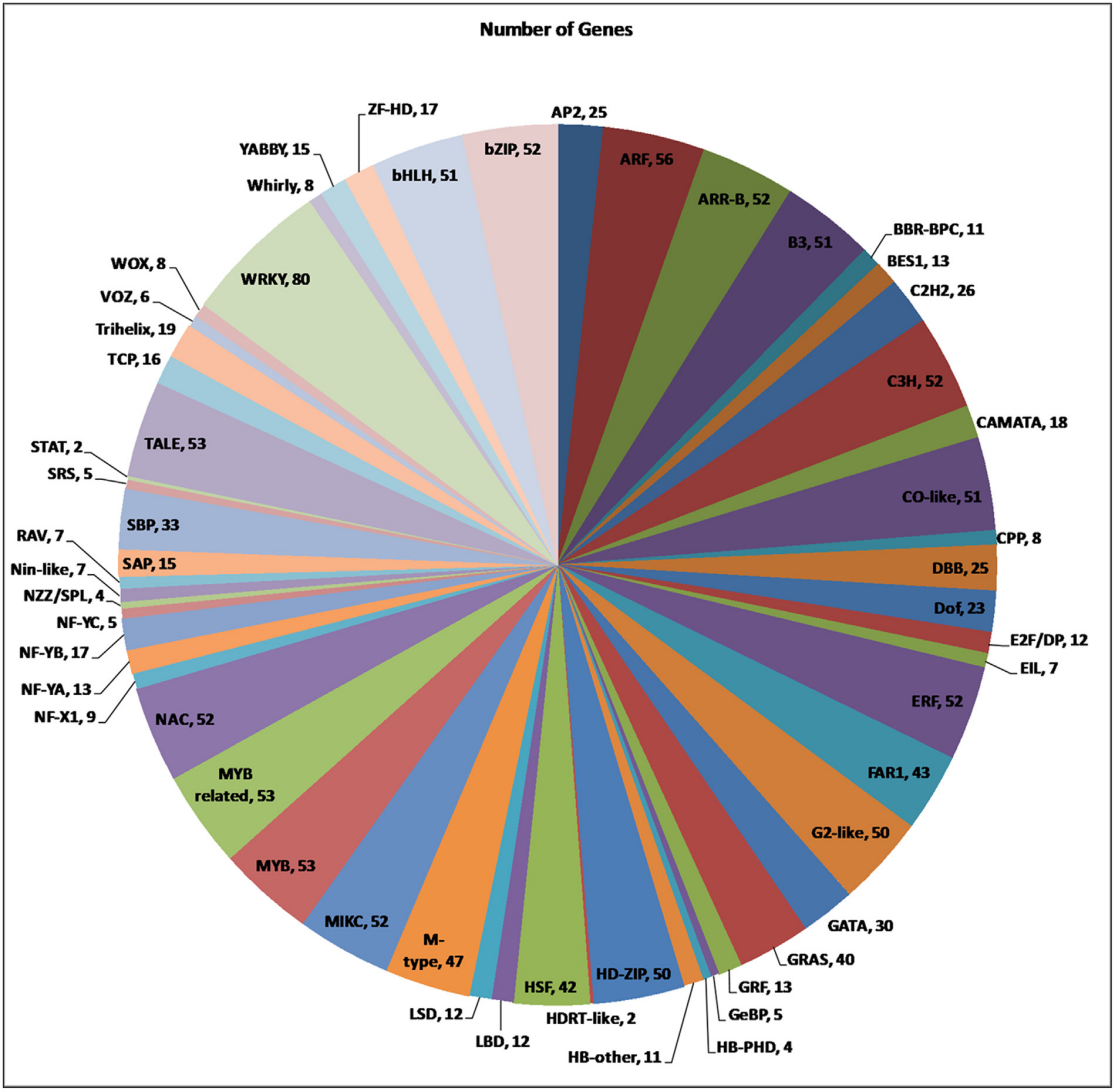
Identified transcription factor details statistics.

We annotated our contigs to the *Arabidopsis thaliana* transcription factor sequences, with the objective of mining classes of transcription factors potentially associated in the differential pattern formation. Transcription factor (sequence-specific DNA-binding factor) is a protein that attaches to specific DNA sequences, thereby controlling the transcription of genetic information from DNA to messenger RNA. Developmental differences and understanding the biology of organ differentiation has been of interest. Various genes involved in organ differentiation, developmental and abiotic and biotic stresses are regulated by transcription factors [49]. Neo-functionalization and sub-functionalization of transcription factors act as key roles in differentiation of plant morphology [50]. We compared the transcription factor between the gynoecious and monoecious lines, to identify candidate factors involved with floral differentiation. Total 58 types of transcription factor associated genes were identified in the two samples of bitter gourd. Based on available transcriptome some important transcription factors *AP2* (25), *ERF* (52), *Dof* (25), *NAC* (52) and *WRKY* (80) associated genes has been successfully identified in bitter gourd (Fig 4).

### DGE Comparisons between the Gynoecious and Monoecious Lines

**Fig 5:**
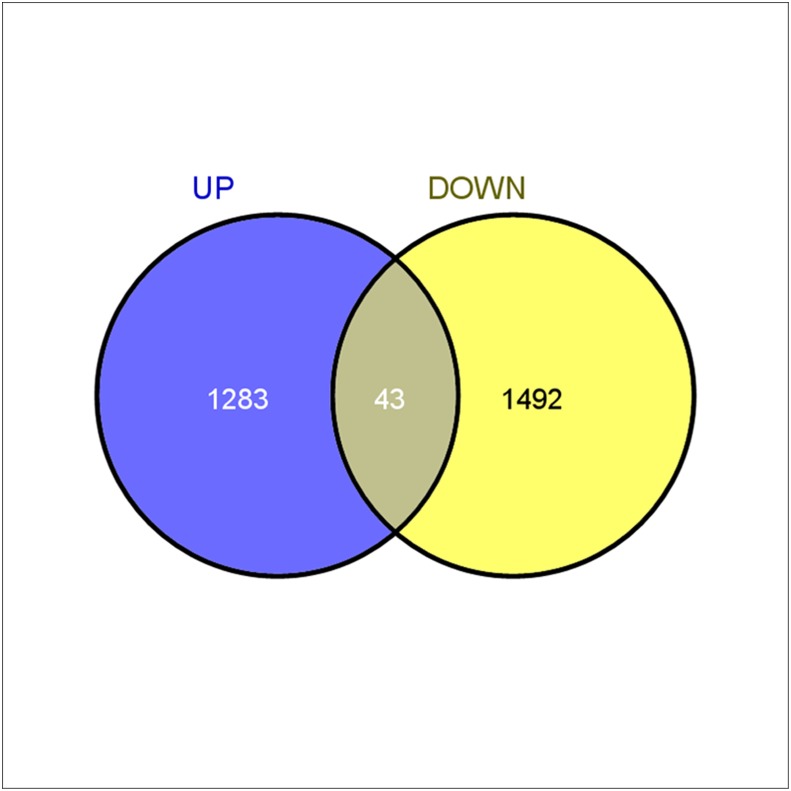
Up and down regulation details of transcripts.

To identify the patterns of gene expression variation in the Mono line (DRAR1) and the Gyno line (Gy323), transcripts count were compared by digital gene expression analysis [51]. A total of 49,685 transcripts out of the 65,535 transcripts were not differentially regulated between the Mono and Gyno lines. In the Gyno line, transcripts corresponding to 6,550 genes were down-regulated, and 9,126 transcripts were up-regulated. From this set our initial focus was on a sub-set of transcripts (with a > = log 2 fold variation with 0.05 Q-significant value). This comprised of a set of 531 transcripts up-regulated in Gyno lines, versus 338 transcripts in up-regulated in Mono line. We also noted 1,492 transcripts were down regulated in Gyno line, and 1,283 transcripts were up regulated while 43 transcripts were neutral (Fig 5), for details, see S6 File.

Effect of gaseous plant growth regulator ethylene, has been demonstrated to affect the gynoecous vs. monoecious phenotypes in melons. Interestingly, of our highly differentially regulated genes were related to hormone signaling and response (S1 and S2 Figs). Auxin and the SAUR/GH3 type factors been involved in gynoecism development [52]. Auxin has also been shown to have critical role in ovule and fruit development [53].

### Identification of SSRs

**Fig 6:**
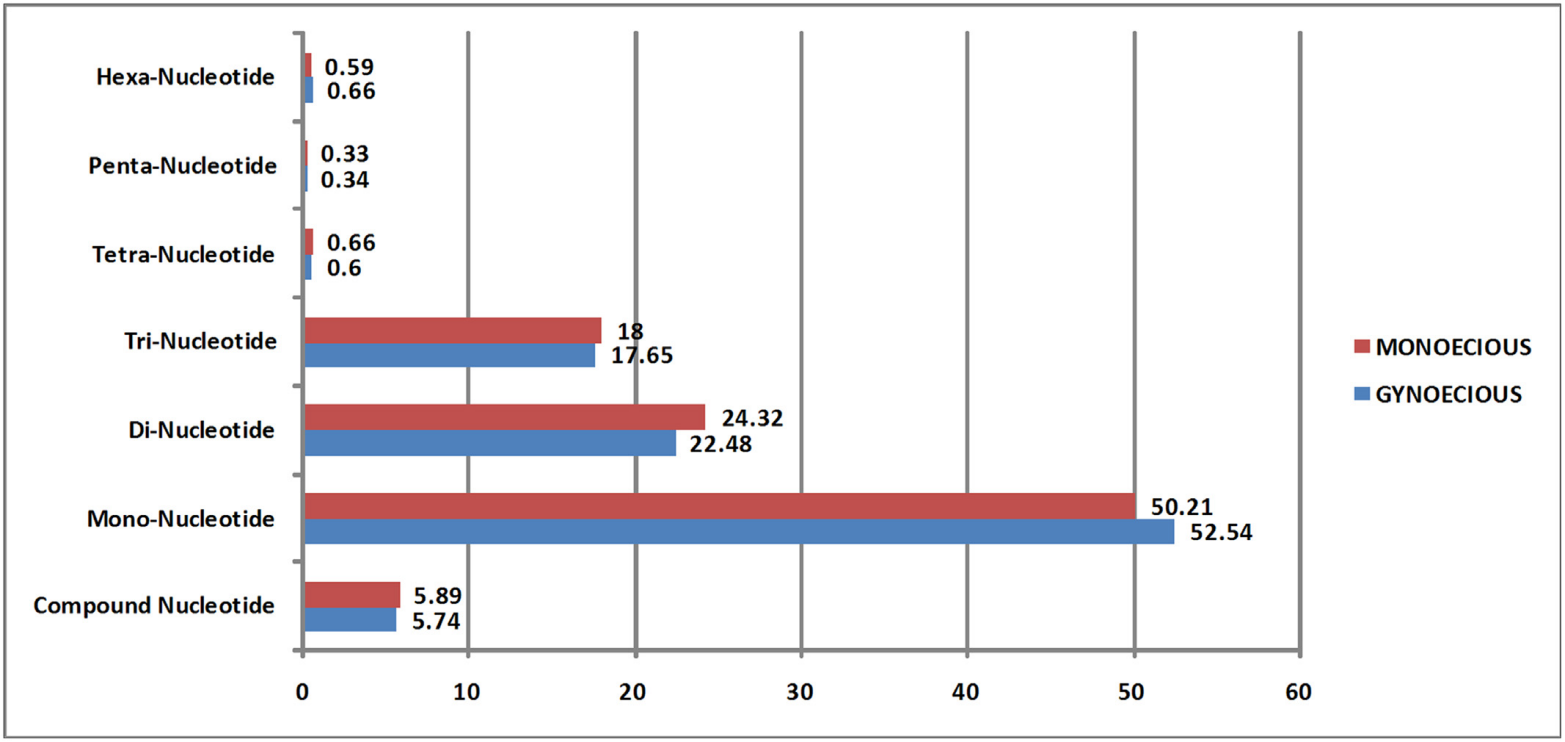
Graph of identified SSR in bitter gourd transcripts.

Total 65,540 sequences for Gyno and 61,490 for Mono were examined for SSR identification using MISA tool. From the total 127,030 contigs, we identified 28,964 SSRs across both lines. A total of 14,471 SSRs where specific to the Gyno line, while we could score 14,493 SSRs for Mono line. Among the SSR, 905 complex repeats were identifies in Gyno line, whereas 882 in Mono lines. Total 94.11% of mono, di, tri, tetra, penta and hexa-nucleotide SSR were present for Mono and 94.27% for Gyno line (Table 2, Fig 6). Based on SSR locator, 2,404 and 2,440 potential SSR markers were identified for Mono and Gyno, respectively (S7 File).

**Table 2:** Statistics of SSR identified in bitter gourd transcripts.

**A. Size of microsatellites and number of repeats**		
**Unit size of microsatellite**	**Minimum number of repeats**	
Mono-nucleotide repeats	10	
Di-nucleotide repeats	6	
tri-, tetra-, penta- & hexa-nucleotide repeats	5,5,5,5	
Maximal number of bases interrupting 2 SSRs in a compound microsatellite	100	
**B. Statistics of microsatellite search for Gyno and Mono lines of bitter gourd**		
**Parameters / SSR motif length**	**Gyno**	**Mono**
Total number of sequences examined	65,540	61,490
Total size of examined sequences (bp)	59,295,384	56,534,088
Total number of identified SSRs	14,471	14,493
Number of SSR containing sequences	11,629	11,659
Number of sequences containing more than 1 SSR	2,253	2,239
Number of SSRs present in complex formation	905	882
Number of SSRs with mono- nucleotides	7,721	8,078
Number of SSRs with di-nucleotide	3,740	3,456
Number of SSRs with tri-nucleotide	2,768	2,713
Number of SSRs with tetra-nucleotide	101	93
Number of SSRs with penta-nucleotide	50	52
Number of SSRs with hexa-nucleotide	91	101

### SNP Detection

**Fig 7:**
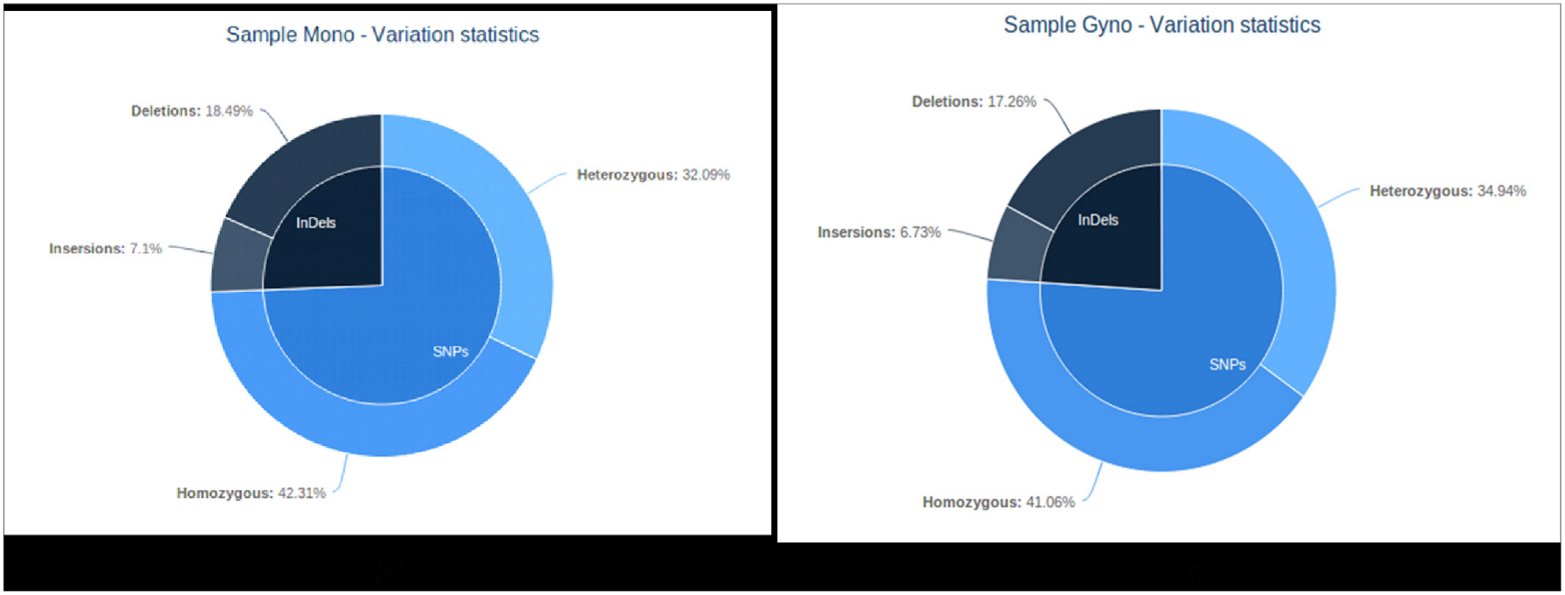
Graphical representation of variation between Gyno and Mono.

SNP from coding regions compared to intergenic regions potentially offer the ability to develop high quality genotyping markers, besides providing insights into functional changes in protein coding domains [54]. To identify expressed allelic variation between the Mono line (DRAR1) and Gyno line (Gy323), variant analysis was performed. We report a total 19,871 SNPs for Mono line and 21,065 for Gyno line. Within these variation 11302 homozygous SNPs were identified in the Mono line and 11381 for Gyno line, respectively. We identified heterozygous SNPs allele, with 8,569 and 9,684 loci reported for Mono and Gyno, respectively. We also identified InDels and a total of 6,836 InDels (1,896 Insertion + 4,940 Deletion) for Mono and 6,650 (1,866 Insertion + 4,784 Deletion) for Gyno were obtained (Fig 7). For details, see S8 File.

### Expression pattern of *McDof* genes in Mono and Gyno lines

**Fig 8:**
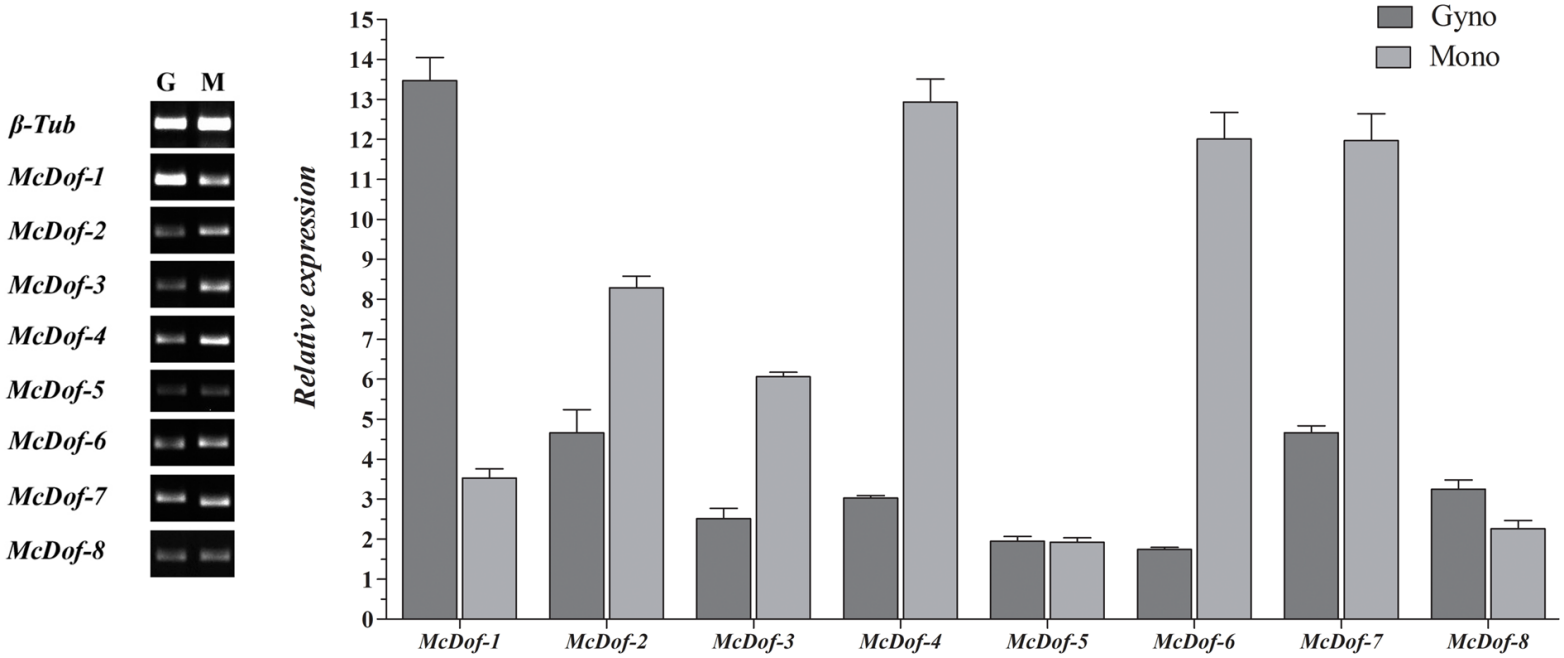
Expression patterns of *McDofs* transcription factors in bitter gourd through qRT-PCR.

The *Dof* (DNA binding with one finger) TF, have domains of bifunctional nature, mediating both DNA-protein and protein-protein interactions. The *Dof* TFs have their role in overall growth and development of the plants including flowering. To narrow the transcripts, for the *Dof* associated genes, we performed a homology search with 30 transcript sequences, to a short-list of 25 candidate sequences, that were further refined to 11 transcripts using BLAST analysis. Phylogentic analysis for 25 identified *Dof* associated genes and for 11 full length genes having complete *Dof* motif signatures, was explored to identify the relationship between these sequences. Further, we could experimentally validate the expression of eight out of these eleven transcripts from the floral RNA though an independent qRT-PCR experiment (Fig 8). As suggested from the NGS data, we were able to demonstrate fold expression changes for these transcripts between the Mono and Gyno line of bitter gourd.

## Discussion

Since the advent of next-generation sequencing, the methods of RNA-seq and bioinformatics analysis workflows have enabled a rapid and detailed understanding of a near complete set of transcripts in a cell of an organism, during their developmental stages and physiological condition [47, 55, 56]. *De novo* assembly of *M*. *charantia* (bitter gourd) transcriptome provides a glimpse of molecular pathways and processes for this important subtropical vine crop of cucurbitaceous family. The transcriptome sequencing of *M*. *charantia* provides opportunities to enable structural and functional study of candidate genes [57–62]. Bitter gourd has been utilized as folk medicines from ancient time to prevent several lethal diseases of mankind, particularly diabetes. It is rich in ascorbic acid, vitamin C and other nutrients that make it a very important crop [63–65]. Nearly all parts of bitter gourd are used for making extracts, powder, capsules, etc.

Several bitter gourd transcripts showed significant similarity to other plants with protein database indicating similarities in their functions. Not unexpectedly we noted sequence identity of transcripts to Cucurbit members, suggesting relatedness. Functional annotation of bitter gourd transcripts revealed significant hits of 51.89% transcripts. However, interestingly 48.11% of total transcripts match to existing sequences in the database, which implicate novel or species-specific functions, possibly connected with the metabolites found in the bitter gourd. These transcripts would enable identification of molecular function, biological process and their cellular components in bitter gourd and related species, which could have medicinal value. Gene ontology statistics showed 55% of transcripts involved in molecular function, 22% in biological process and 23% in cellular component. These functional categories can provide a clue towards studies on specific pathways and their associated functions. Moreover, based on available data, one can correlate the gene-gene network at signal transduction pathways level as well as gene-family level. The *in silico* study may be a step towards unraveling biological phenomena through sequential, structural and functional genomics studies for crop improvement and nutritional quality purposes.

In bitter gourd hybrid seeds are normally produced utilizing manual pollination method which is time and labor intensive and expensive. Gynoecism is an advantageous trait for hybrid seed production and has been extensively applied in cucumber breeding programs [66, 67]. In related melons the sexual phenotypes can be modulated with extrageneous agents such as ethylene, however these mechanisms have not been explored in bitter gourd. For cucurbits such as pumpkins, squash, *Luffa*, and melons, the genome and transcriptome sequencing projects are in progress. Genome sequence of closely related cucurbit, *C*. *sativus is* published and gives opportunities for functional study [68]. Two genotypes, gynoecious (Gyno) line Gy323 and monoecious (Mono) line DRAR1 of bitter gourd were selected for transcriptome sequencing. These lines differ phenotypically in the floral organ development, with the absence of male flowers in the Gy323 line [16]. We compared the transcript expression profiles of these lines. Based on *de novo* transcriptome assembly 95.91% reads for monoecious and 95.18% reads for gynoecious were assembled with ~919 and ~904 average contig length, respectively. The coverage of *Momordica charantia* transcripts was comprehensive and exhibited high quality and length of transcripts obtained. GC content analysis revealed that the GC% distribution within 40–49 with a maximum 80% of contig hits similar to other plant species. The GC content analysis provides insights into several aspects including evolution, thermo stability and gene regulation [69]. In this study,differential gene expression analysis of the annotated transcripts identifies a class of plant hormone response pathways that are differentially regulated, and could have implication in the development of the sexual phenotypes. The ability to combine expression analysis with the genetic mapping studies will enable identification of the key players in the hormonal regulation of sexual characters in cucurbits.

In case of *M*. *charantia*, limited information on SSR markers has been reported [23, 70, 71], however to date, little information exists on the SNPs. SSR and SNP variation enable development of population studies, kinship, and classification of individuals based on haplotypes [72]. Further these tools can facilitate identification of synteny and gene duplication/deletion events across cucurbit members [73–75]. Variations in the DNA sequences of plant genome can affect how plants develop diseases and respond to pathogens, chemicals and other agents, besides being deployed as molecular breeding tools for trait association or molecular breeding [76, 77] and disease management [78, 79].

Based on transcriptome sequencing, maximum 2,440 SSR primers for Gyno and 2,404 for Mono were designed. Patterns of SSR variation between the Gyno Gy323 line and the Mono DRAR1 line can be used to screen and develop markers to type and identify lines for the gynoecism trait.

In our analysis, we identified a large number of SNP variants, a total of 19,871 SNPs for Mono and 21,065 for Gyno were detected. Number of homozygous SNPs specific for the DRAR1 Mono line was 11,302, while 11,381 SNP were identified for the Gy323 Gyno line.

Among the highly differentially regulated genes, several transcripts involved in processes such as signaling and development were expressed in the Gyno line compared to the Mono line (see S9 File). Out of 11 full length *Dof* associated genes, we successfully validated 8 transcripts (*McDof-1* to *McDof-8*) and were able to demonstrate fold expression changes for these transcripts between the Mono and Gyno line of bitter gourd (Fig 8). We also conducted pathway analysis of the set of differential genes for the Mono and Gyno line to identify the set of genes enriched for biochemical pathways. These candidate genes suggest involvement of developmental and signaling on line specific differential development programs. Most crucially we report on the differential expression of genes orchestrating the hormone response pathways. Exploring the master regulators for these pathways, and exploring the comparative response across cucurbits demonstrating sexual heteromorphy could provide deep insights into breeding and engineering high value traits into bitter gourd.

## Conclusions

Comprehensive transcriptomics enables creation of molecular resources for an important cucurbit member and enables identification of candidate genes, besides generation of functional molecular markers. SNP markers will facilitate higher resolution polymorphism identification for breeding improved bitter gourd populations, though marker assisted breeding. Based on available resources, pathway related genes can be identified using comparative genomics. The present transcriptomics analysis provides valuable biological information for candidate genes and transcripts in bitter gourd and the transcriptome sequences may provide better insights into the biology of *M*. *charantia*.

## Supporting Information

S1 Fig Heat map showing hierarchical clustering of hormone biosynthesis pathway related genes in two samples of bitter gourd (Gyno pool and Mono pool). Dark red color expressing higher fold changes of expressed genes as compared to green color.
(TIF)

S2 Fig Plant hormone signal transduction pathway mapped with Gyno and Mono transcripts. (TIF)

S1 File Primary analysis details. (ZIP)

S2 File SSR details and markers. (ZIP)

S3 File Variation statistics. (ZIP)

S4 File Similarity analysis report. (ZIP)

S5 File Annotation report. (ZIP)

S6 File KOG Annotation report. (ZIP)

S7 File Digital expression analysis report with clustered transcripts. (ZIP)

S8 File Analysis report. (ZIP)

S9 File Pathway analysis report. (ZIP)

S1 Table Primers used in qPCR study, for the validation *McDof* Transcription Factors in bitter gourd. (DOCX)

S2 Table Important software along with version and parameters used for transcriptomic study in bitter gourd. (DOCX)
