# Supplementary figures and images for: De Novo Assembly of Bitter Gourd Transcriptomes: Gene Expression and Sequence Variations in Gynoecious and Monoecious Lines
Source: PLoS One. 2015 Jun 5;10(6):e0128331. doi: 10.1371/journal.pone.0128331 (PMC4457790; doi:10.1371/journal.pone.0128331)

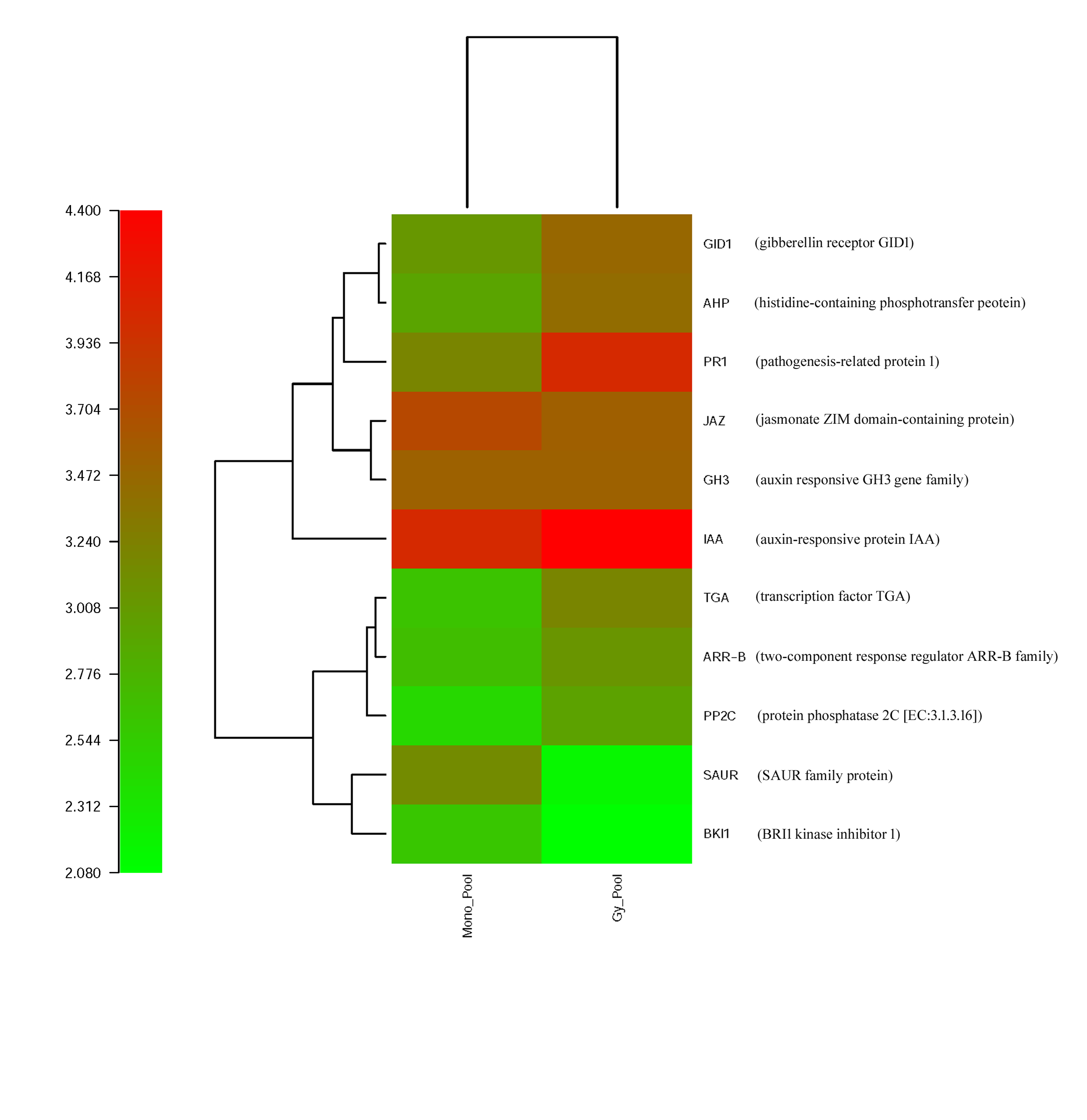

Supplement: S1 Fig — Dark red color expressing higher fold changes of expressed genes as compared to green color. (TIF) [file pone.0128331.s001.tif]

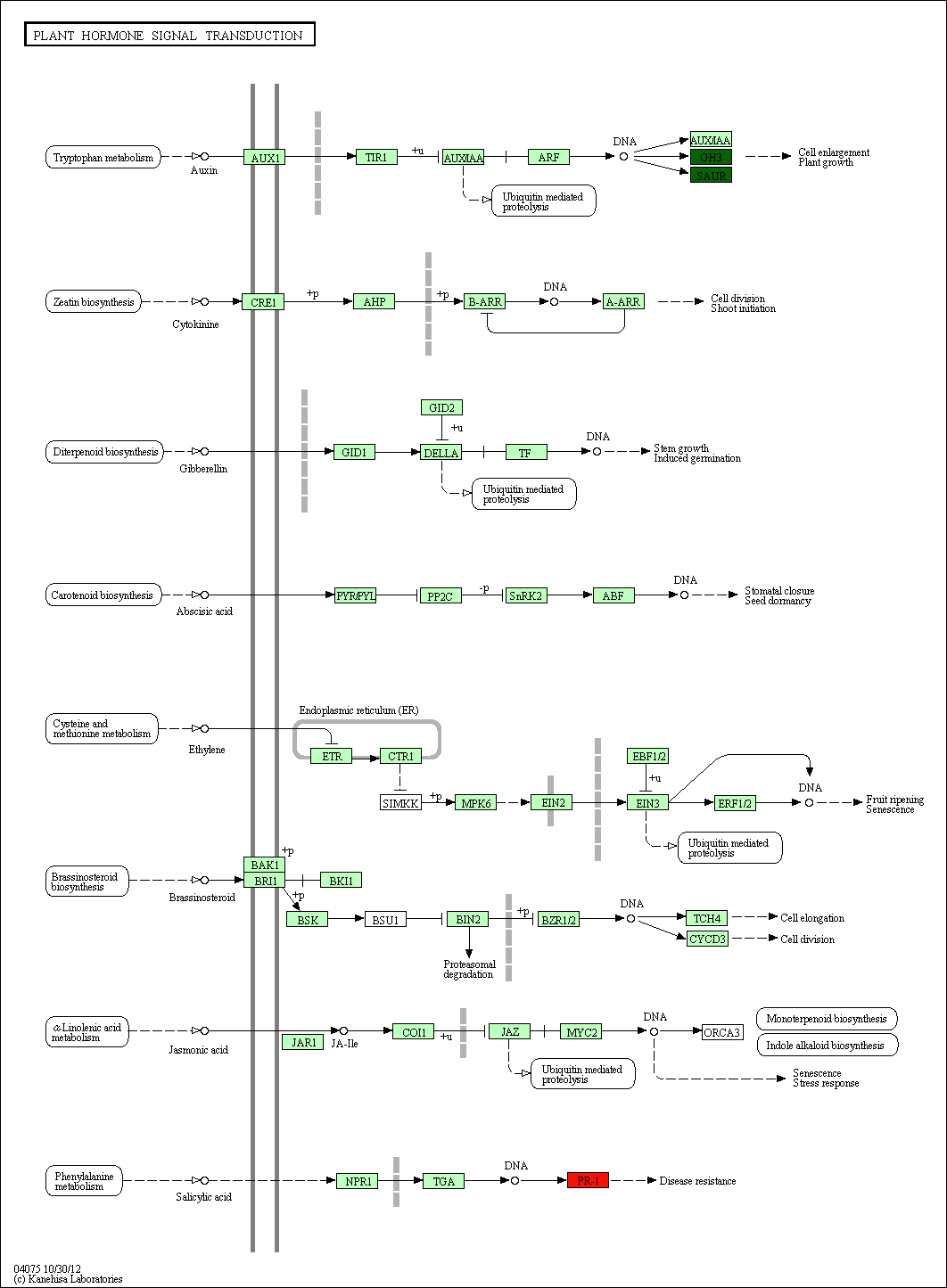

Supplement: S2 Fig — (TIF) [file pone.0128331.s002.tif]
