# Supplementary material for: De Novo Assembly of Bitter Gourd Transcriptomes: Gene Expression and Sequence Variations in Gynoecious and Monoecious Lines
Source: PLoS One. 2015 Jun 5;10(6):e0128331. doi: 10.1371/journal.pone.0128331 (PMC4457790; doi:10.1371/journal.pone.0128331)
Supplement: S1 Table — (DOCX) [file pone.0128331.s012.docx]

**Supplementary Table 1. Primers used in qPCR study, for the validation *McDof* Transcription Factors in bitter gourd**

| **Abbreviation** | **Primer sequence (5′-3′)** | **Description** |
| --- | --- | --- |
| *McDof-1* | GGCTGCAGGAAGAACAAAAG | *M. charantia* *McDof-1* primer, forward |
| *McDof-1* | GTGGTTTCAAAGGCCAAAGA | *M. charantia* *McDof-1* primer, reverse |
| *McDof-2* | CCCGATCTCCATGATCAGTT | *M. charantia* *McDof-2* primer, forward |
| *McDof-2* | CCTTTCCCTTCCTCAACTCC | *M. charantia* *McDof-2* primer, reverse |
| *McDof-3* | CTTCTCACAGCTTGCTGCTG | *M. charantia* *McDof-3* primer, forward |
| *McDof-3* | CCGTCTGATCTTGATGTTGATG | *M. charantia* *McDof-3* primer, reverse |
| *McDof-4* | TCCTCAATTTTGGCTCAGATTC | *M. charantia* *McDof-4* primer, forward |
| *McDof-4* | CCGGTGGCTGTGAAGTTATT | *M. charantia* *McDof-4* primer, reverse |
| *McDof-5* | CACTGCAGCTGATTATAACCCTT | *M. charantia* *McDof-5* primer, forward |
| *McDof-5* | CCAATTTCCCTCCATTCTGA | *M. charantia* *McDof-5* primer, reverse |
| *McDof-6* | GAATCCCAGATTTCCTCCG | *M. charantia* *McDof-6* primer, forward |
| *McDof-6* | CTTCATTATCGCCTCCCAAA | *M. charantia* *McDof-6* primer, reverse |
| *McDof-7* | AGAGGCTCTTCAAGCTGCAC | *M. charantia* *McDof-7* primer, forward |
| *McDof-7* | TCCTGGTTCCATTCAAGACC | *M. charantia* *McDof-7* primer, reverse |
| *McDof-8* | CATGCTGATTTCTCCAATTATCAA | *M. charantia* *McDof-8* primer, forward |
| *McDof-8* | CCCAGAAAATTCCTCGACAA | *M. charantia* *McDof-8* primer, reverse |
| *β-Tub* | CATCTTCCACCTTTACACCCTG | *M. charantia* *β-Tubulin* primer, forward |
| *β-Tub* | CCATGTATCAATCAAACACTCC | *M. charantia* *β-Tubulin* primer, reverse |
