# Supplementary material for: De Novo Assembly of Bitter Gourd Transcriptomes: Gene Expression and Sequence Variations in Gynoecious and Monoecious Lines
Source: PLoS One. 2015 Jun 5;10(6):e0128331. doi: 10.1371/journal.pone.0128331 (PMC4457790; doi:10.1371/journal.pone.0128331)
Supplement: S2 Table — (DOCX) [file pone.0128331.s013.docx]

| **Step** | **Software Used** | **Parameters** |
| --- | --- | --- |
| Assembly | velvet_1.1.07 | Minimum contig length of 100, |
|  | oases_0.2.01 | Default |
| Clustering | cd-hit-v4.5.4-2011-03-07 | 95% percent identical sequences were merged |
| Annotation | ncbi-blast-2.2.26+ | Default |
| Misa | Perl Script | Single repeat should occur 10 times, di-nucleotide repeats should occur 6 times, and 3 to 6 base repeats to occur at least 5 times. Any SSRs which were separated by a distance of 100bp were considered as compound SSRs. |
| Alignment Tool used | bowtie2-2.0.0-beta5 | Default |
| Sequence quality check | SeqQC V 2.0 | Default |
| KOG Analysis | KOG db | Cutoff: 30% identify and 30 % subject coverage. |
| Variations detection tool | Samtools 0.1.7a | Default |
| DGE | DESeq | Default |

**Supplementary Table 2. Important software along with version and parameters used for transcriptomic study in bitter gourd**
