# Supplementary material for: De Novo Assembly of Bitter Gourd Transcriptomes: Gene Expression and Sequence Variations in Gynoecious and Monoecious Lines
Source: PLoS One. 2015 Jun 5;10(6):e0128331. doi: 10.1371/journal.pone.0128331 (PMC4457790; doi:10.1371/journal.pone.0128331)
Supplement: S8 File — (ZIP) [file pone.0128331.s010.zip › Supplementary 8/Analyzed Data.doc]

(Gyno)

(Monoo0o)

**Fig. 1 Graph showing organisms vs contigs GC% distribution of Bittergourd samples.**

Fig. 2 Sequence similarity of Bitter gourd transcript to different organisms.

Fig. 3 Functional annotation graph of cellular components for gyno & mono samples

Fig. 4 Functional annotation graph of biological process for gyno& mono samples

Fig. 5 Functional annotation graph of molecular function for gyno & mono samples

Fig 6. Rooted phylogenetic tree using UPGMA

Table 1: Comparative table between *Momordica charantia* and *Cucumis sativus* with Identity, Positivity and Gap percentage.

| S.N. | Momordica charantia_Dof_Primary Accession No. | Similar gene id in Cucumis sativus | Identities % | Positives % | Gaps (Insertion + Deletion) % |
| --- | --- | --- | --- | --- | --- |
| 1. | Mc_23_GANG01044217.1_ Momordica_charantia | Cucsa.172400.1\|Cucumis_sativus\|Dof\|Cucsa.172400.1 | 49% | 56% | 18% |
| 2. | Mc_15_GANG01010921.1_ Momordica_charantia | Cucsa.010690.1\|Cucumis_sativus\|Dof\|Cucsa.010690.1 | 84% | 87% | 3% |
| 3. | Mc_22_GANG01007457.1_ Momordica_charantia | Cucsa.046960.1\|Cucumis_sativus\|Dof\|Cucsa.046960.1 | 77% | (80%) \| | (11%) |
| 4. | Mc_13_GANG01026112.1_ Momordica_charantia | Cucsa.213150.1\|Cucumis_sativus\|Dof\|Cucsa.213150.1 | 66%) | 76% | 15%) |
| 5. | Mc_14_GANG01021916.1_ Momordica_charantia | Cucsa.338050.1\|Cucumis_sativus\|Dof\|Cucsa.338050.1 | (85% | 89% | 2% |
| 6. | Mc_19_GANG01002159.1_ Momordica_charantia | Cucsa.112810.1\|Cucumis_sativus\|Dof\|Cucsa.112810.1 | 59% | (69% | 16%) |
| 7. | Mc_12_GANG01013460.1_ Momordica_charantia | Cucsa.197750.1\|Cucumis_sativus\|Dof\|Cucsa.197750.1 | 84% | 89%) | 0% |
| 8. | Mc_11_GANG01003701.1_ Momordica_charantia | Cucsa.338740.1\|Cucumis_sativus\|Dof\|Cucsa.338740.1 | 81% | 86% | (2% |
| 9. | Mc_10_GANG01045981.1_ Momordica_charantia | Cucsa.284990.1\|Cucumis_sativus\|Dof\|Cucsa.284990.1 | 76% | 80%) | (4%) |
| 10. | Mc_18_GANG01012575.1_ Momordica_charantia | Cucsa.113470.1\|Cucumis_sativus\|Dof\|Cucsa.113470.1 | 57%) | 67% | 0% |
| 11. | Mc_17_GANG01012686.1_ Momordica_charantia | Cucsa.198560.1\|Cucumis_sativus\|Dof\|Cucsa.198560.1 | 71%) | 76%) | 0% |
| 12. | Mc_8_GANG01040334.1_ Momordica_charantia | Cucsa.032330.1\|Cucumis_sativus\|Dof\|Cucsa.032330.1 | 82%) | (88% | (4%) |
| 13. | Mc_9_GANG01008895.1_ Momordica_charantia | Cucsa.032330.1\|Cucumis_sativus\|Dof\|Cucsa.032330.1 | 82% | 88% | 4% |
| 14. | Mc_3_GANG01040568.1_ Momordica_charantia | Cucsa.259380.1\|Cucumis_sativus\|Dof\|Cucsa.259380.1 | 87% | 88% | 6% |
| 15. | Mc_6_GANG01024718.1_ Momordica_charantia | Cucsa.304130.1\|Cucumis_sativus\|Dof\|Cucsa.304130.1 | 67% | 74% | 11% |
| 16. | Mc_4_GANG01020009.1_ Momordica_charantia | Cucsa.202290.1\|Cucumis_sativus\|Dof\|Cucsa.202290.1 | 48% | 54% | 27% |
| 17. | Mc_5_GANG01031748.1_ Momordica_charantia | Cucsa.202290.1\|Cucumis_sativus\|Dof\|Cucsa.202290.1 | 53% | 59% | 21% |
| 18. | Mc_1_GANG01025632.1_ Momordica_charantia | Cucsa.046290.1\|Cucumis_sativus\|Dof\|Cucsa.046290.1 | 77% | 78% | 3% |
| 19. | Mc_2_GANG01043741.1_ Momordica_charantia | Cucsa.052650.1\|Cucumis_sativus\|Dof\|Cucsa.052650.1 | 88% | 92% | 3% |
| 20. | Mc_16_GANG01001816.1_ Momordica_charantia | Cucsa.207460.1\|Cucumis_sativus\|Dof\|Cucsa.207460.1 | 76% | 82% | 4% |
| 21. | Mc_21_GANG01039249.1_ Momordica_charantia | Cucsa.098430.1\|Cucumis_sativus\|Dof\|Cucsa.098430.1 | 77% | 85% | 6% |
| 22. | Cucsa.046300.1\|Cucumis_sativus\|Dof\|Cucsa.046300.1 | Mc_7_GANG01043079.1_ Momordica_charantia | 57% | 65% | 15% |
| 23. | Mc_24_GANG01015920.1_ Momordica_charantia | Cucsa.126810.1\|Cucumis_sativus\|Dof\|Cucsa.126810.1 | 59% | 69% | 13% |
| 24. | Mc_20_GANG01044062.1_ Momordica_charantia | Cucsa.126810.1\|Cucumis_sativus\|Dof\|Cucsa.126810.1 | 70% | 82% | 7% |
| 25. | Mc_25_GANG01044448.1_ Momordica_charantia | Cucsa.341290.1\|Cucumis_sativus\|Dof\|Cucsa.341290.1 | 100% | 100% | 0 |
